# Development of dry reagent chemistry for the clinical laboratory

**DOI:** 10.1155/S1463924681000205

**Published:** 1981

**Authors:** Adam Zipp

**Affiliations:** Blood Chemistry Laboratory Ames Divison Miles Laboratories Eikhart Indiana USA


### Conclusions

It appears that on balance, these two systems are similar in terms of advantages and disadvantages. Each seems to have emphasised the more important characteristic for its potential application. Thus, the multilayer film tests seem to be being directed to large laboratories where high precision is important and storageability less so. The impregnated fibre systems, on the other hand, seem to be being directed toward the small laboratory, the physician's office, or the emergency room, where convenience, low space requirements, and simplicity are important. Unquestionably, as dry reagent technology matures, each will borrow from the other to achieve the best features of both. One should bear in mind that the technologies, as they are currently configured, are only the beginning. They may, however, signal the future of clinical chemistry. If successful, they certainly will bring a new and desirable level of analytical standardisation to the industry and provide the medical community with a more universal basis for communication. Large scale manufacturing with its associated quality control methods should provide a new level of analytical consistency. Laboratory regulation should become simplified. Finally, the new technology should enhance cost effectiveness in health care because it provides capability for test selectivity, because of its ease of use, and because individual self-contained tests allow for little material waste.

REFERENCES

[1] Kubelka P. and Munk F., Z. (1931) *Tech. Physik.*, **12**, 593
[2] Williams F. C. and Clapper F. R., (1953) *J. Opt. Soc. Amer.*, **43**, 595
[3] Curme, H.G., et al, (1978) *Clin. Chem.*, **24**, 1335.
[4] Eder T. W., Abst., X Intl. Cong. Clin. Chem., Mexico City, Feb., 1978
[5] McGlothlin, C.D., Abst. No. 375, 30th Natl. Mtg., A.A.C.C., San Francisco, July, 1978
[6] Wu, T. and Dappen, M., U.S. Patent 4,069,017

### Development of dry reagent chemistry for the clinical laboratory*

Adam Zipp
*Blood Chemistry Laboratory, Ames Divison, Miles Laboratories, Eikhart, Indiana, USA.*

### Introduction

The Ames Division of Miles Laboratories has, for the past several years, been actively involved in the development of a quantitative serum chemistry system. Initially, the system will consist of the instrument, a reflectance photometer called the Seralyzer, along with five solid phase reagents: glucose, BUN, uric acid, bilirubin, and LDH. Reagents to analyse for cholesterol and triglycerides are also under development. The technology necessary for the development of this system stems from the development by Ames of urine strip products. The knowledge obtained from the development of the Dextrostix/Eyetone system for the quantitative analysis of whole blood glucose also contributes. The company has however, extended this technology to include a host of serum metabolites as well as enzymes. A schematic of the solid phase reagent strip is shown in Figure 1. This strip contains a cellulose matrix into which is impregnated those reagents necessary for a given clinical chemical determination. After the impregnation process, the matrix is dried and bonded with a special adhesive layer onto a plastic support which allows for ease of insertion onto and removal from the instrument. The philosophy adopted during the development of this system is to employ well-known methodologies wherever possible. The use of cellulose provides a good deal of flexibility in this regard so that it is possible to impregnate a host of different materials into the matrix, even under extreme conditions. For example, serum bilirubin is quantitated with a diazonium coupling reaction which is carried out at pH near 1. Similarly, the solid phase BUN test is carried out in a cellulose matrix which is a 50% cation exchange resin so that very low pH conditions are also achieved here.

*Paper presented at the Analytica 80 Symposium on Dry Reagent Chemistry in Munich, April, 1980.*

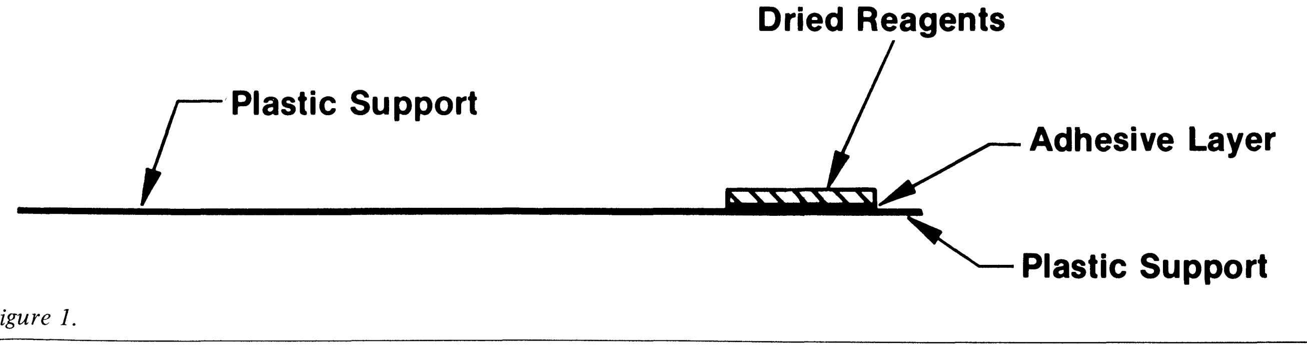

*Figure 1.*

It is also possible to incorporate into this matrix the reagents necessary for a number of coupled reactions in such a way that chemical interactions do not take place until the reagents are rehydrated. In this regard, the triglycerides reagent pad contains the reagents necessary to carry out a reaction sequence which includes four enzymatically coupled reactions. Furthermore, it is also important to recognize that the impregnation and drying process which is used in the system produces self-contained reagent strips which are unusually stable, providing room temperature stability in excess of one year even for strips containing NADH which is highly unstable in wet systems.

Besides this excellent stability characteristic, the solid phase format completely eliminates the need for the normal manipulative procedures encountered with wet reagent systems. The user need only deposit 30 $\mu$l of sample onto the pad surface. This sample hydrates the reagent area, and activates the reagents. This activation results in the production of colour which is proportional to the concentration of the particular analyte in the sample.

### Detection system

In order to produce a quantitative system, it is necessary to measure the intensity of this colour accurately. Unlike wet systems where colour intensity is measured with absorption spectroscopy, the measurement technique most suitable for this system is reflectance spectroscopy. The basis of this measurement technique is shown in Figure 2. Figure 2(b) illustrates the simplest way of making reflectance measurements. The sample surface is illuminated at some arbitrary angle and the reflectance measured by placing the detector at some other arbitrary angle. This technique is, however, generally inadequate for our purposes. A better way for measuring the reflectance of a surface is with an integrating sphere, which is shown in Figure 2(a). This is simply a closed sphere with a highly reflecting surface into which the sample is placed. In this configuration, light incident on the sample that is not immediately reflected to the detector will be multiply reflected about the sphere surface until it finally reaches the detector or is absorbed by the sphere wall. In the Ames system, the intensity of light which is reflected from the pad surface is proportional to the amount of colour which is formed on the reagent pad. As mentioned above, this colour is related to the concentration of analyte under consideration.

The development effort was obviously centred around the mating of these two components – the measurement technique and the solid phase reagent. With this in mind, it was important that the instrument be designed so that system performance could be maximised. This required such instrumental factors as precise temperature control, reproducibility of pad placement on the instrument table, a high intensity light source so that the reflectance signal could be maximised, microprocessor control as well as a number of optical considerations. Furthermore, it was necessary that the instrument and the reagent be developed in unison so that important system problems could be recognised and corrected as early as possible.

### Instrument design

A schematic of the final instrument optical system is shown in Figure 3. The instrument utilises an integrating sphere to collect the light which is reflected from the surface of the reagent pad. The light source is a Zenon flash tube which produces a high intensity flash containing light over the entire spectral region. In particular, this source provides a high light intensity at 340 nm, so that enzyme reactions can be carried out in the UV spectral region. The reagent strip itself sits on a thermostated table which is pushed into the integrating sphere after sample application. The environment of the sphere is also thermostatically controlled so that the reaction conditions can be maintained constant. Projecting into the sphere is a collimating device which collects the light reflected from the pad surface and directs it through an interference filter to a solid state detector. Also projecting into the sphere is the reference detector port which directs light scattered from the sphere wall to the reference detector. The raw reflectance is then the ratio of the sample and reference detector signals. This procedure eliminates error in reflectance reading due to changes in light intensity from flash to flash. The sapphire window which covers the sample and reference detector ports is used to minimise evaporation from the pad surface. The interference filter which covers the detector ports is contained in a plug-in module which is specific for each serum test. This module and its relationship to the instrument optical system is shown in Figure 4. This figure also shows the integrating sphere with the table and strip inside of it. This module contains not only the appropriate filter for a given test, but also a read-only memory chip which contains the instructions necessary for proper processing of the reflectance signals. For example, such things as the number of reflectance readings as well

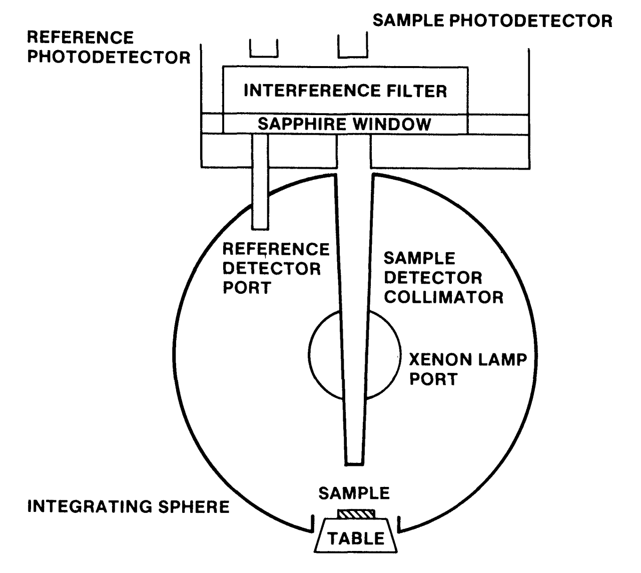

*Figure 3.*

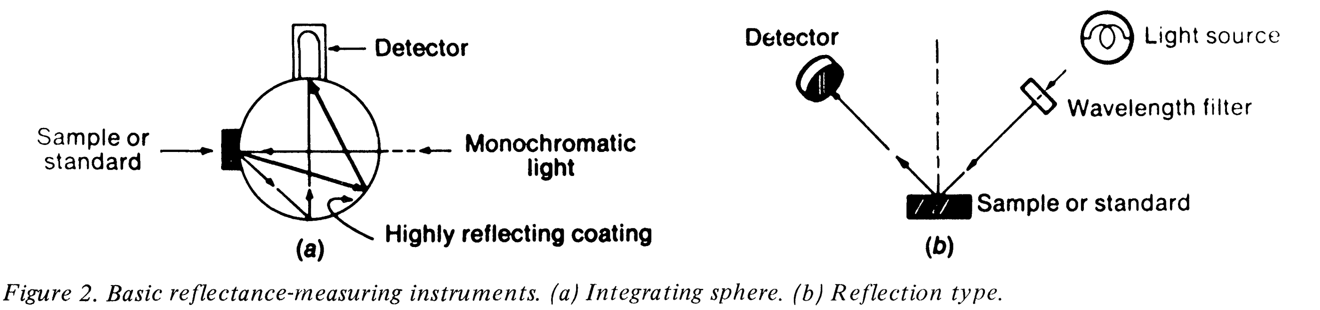

*Figure 2. Basic reflectance-measuring instruments. (a) Integrating sphere. (b) Reflection type.*

as the time intervals between readings is programmed into this chip and communicated to the instrument microprocessor.

Therefore, with microprocessor control of the instrument functions a good deal of flexibility is allowed in the analysis of the reflectance data so that the performance of each test can be maximised. One of the ways in which this flexibility is used is illustrated in Figure 5. This shows some reaction profiles for the solid phase LDH test. In this test, LDH activity is quantitated by measuring the rate of disappearance of NADH at 340 nm. The total test time is 120 seconds. At low LDH activity, the change in percent reflectance is linear over the entire course of the reaction. As the LDH activity increases, however, the reaction profiles become non-linear as the reaction proceeds, presumably because of product inhibition and/or substrate depletion. In order to achieve maximum precision for this system it is important that the total change in measured reflectance is a maximum. This is especially important when compounds such as NADH with relatively small extinction coefficients are used as the colour generating species. For the profiles shown, it is desirable to use the data for the full two minutes at low LDH activities to obtain the maximum reflectance change. On the other hand, at high LDH activities, it is not possible to include data for the full 120 seconds since the profiles become non-linear. It was, therefore, necessary to write the mathematical algorithm for this test such that starting at t=0 only the maximum change in reflectance with time is used in the actual data calculation. When the reaction profiles begin to deviate from linearity, therefore, the slope of the line will also decrease and further data points will not be used in the slope calculation. In this way, data for the full 120 seconds of the reaction corresponding to the largest change in reflectance will be used at low LDH activities. It is, of course, not necessary to use data for this full time period at high LDH activities because the changes are large.

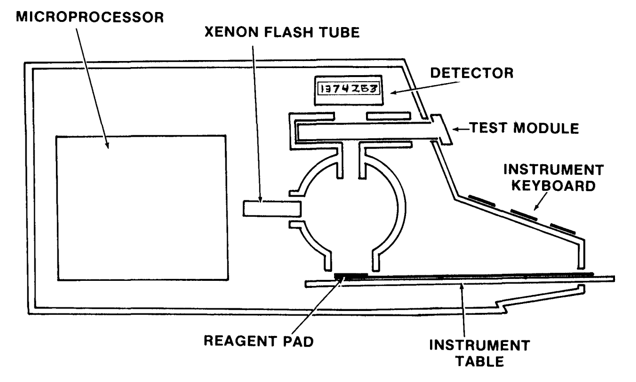

*Figure 4.*

In order to account for variations in reagent reactivity from lot to lot or as the strips age, this system utilises a two-point "live" calibration procedure to establish a linear relationship between reflectance (or an appropriate transformation thereof) and analyte concentration. "Low and high" calibration solutions of known concentration are used to establish this relationship. A typical calibration curve for LDH is shown in Figure 5(b). The d% R/dt values at 100 to 400 IU/L obtained from the curves in Figure 5(a) were used to generate this line. The point at low LDH activity was, therefore, obtained from the change of reflectance with time over the full 120 seconds of the test; whereas for the high calibration point which would be at about 400 IU/L, only about 40 seconds of data are actually used in the determination. The calibration procedure is identical for each test and each possesses its own mathematical algorithm.

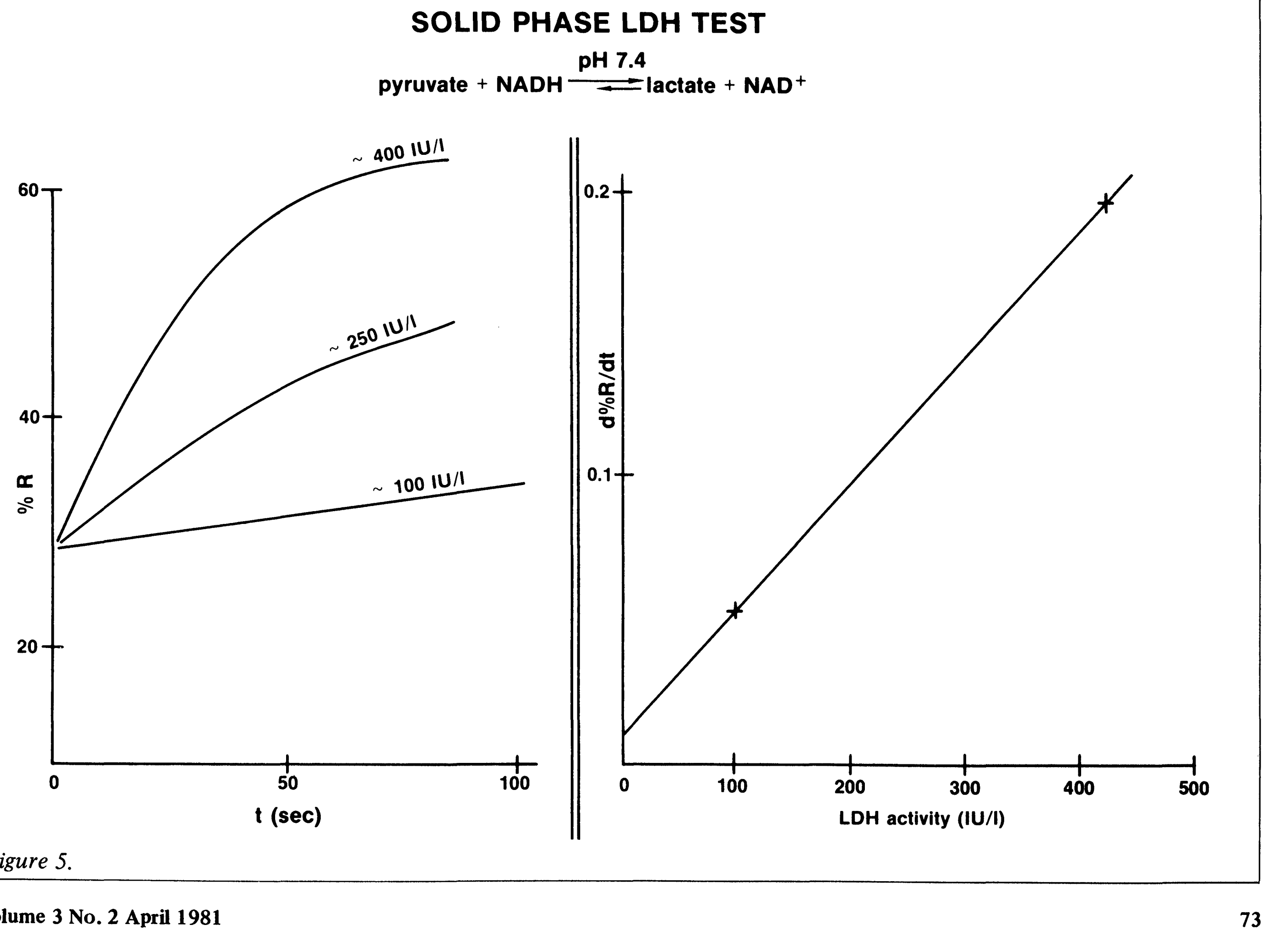

*Figure 5.*

With microprocessor control of instrument functions, it is also possible to build into this system a series of checks and balances on system performance so that any malfunctions or operator error can be easily detected. For example, upon completion of the calibration, the instrument microprocessor checks the calibration slope to determine its validity. If outside the required specification, an error message is given. Furthermore, such conditions as improper sample dilution, unreactive reagent, loss of instrument thermal control or improper operating procedures will be recognised by the instrument and an appropriate error message flashed on the instrument display.

A summary of the chemistries, test times, reaction types and instrument wavelength for the seven tests mentioned earlier is given in Figure 6. All of these tests utilise state of the art methodologies with test times ranging from about 1 to 2 minutes, depending on the test. Glucose, uric acid and cholesterol utilise enzyme catalysed oxidation reactions to produce hydrogen peroxide which oxidises an indicator system to produce a coloured complex. As mentioned above, the bilirubin test utilises a diazonium coupling reaction and the LDH test employs the oxidation of NADH at 340 nm. The triglycerides tests employs three enzymatically coupled reactions to produce $NAD^+$. This $NAD^+$ reacts with the tetrazolium salt INT in the presence of diaphorase to produce a coloured product.

### Instrument performance

A system is described above which consists of a solid phase reagent strip and an instrument which was specifically designed for such a reagent format. The performance data obtained from such a system is described here. Figure 7 shows some precision data for a representative rate test BUN, enzyme test LDH and end point test cholesterol. These data represent overall precision and include both within-run and between-run components. The between-run component includes, of course, the effects of multiple calibrations. The overall coefficients of variation in Figure 7 are calculated as the square root of the sum of the squares of these two components, which are approximately equal for the system.

The coefficients of variation given average around 5% for each analyte given over the entire useful range. These data clearly indicate precision which is more than adequate for clinical efficacy. Furthermore, these data are comparable to precision obtained with state-of-the-art wet methods given in a recent survey by Ross and Frazer [1]; Figures 8-10 show correlation data for these serum components obtained

| Test | Chemistry | Time | Type | λ |
|---|---|---|---|---|
| Glucose | glycose oxide/peroxidase | 60 sec | Rate | 640 nm |
| BUN | o-phthaladehyde/HTBQ | 75 sec | Rate | 620 nm |
| Bilirubin | diazonium coupling | 75 sec | End Point | 560 nm |
| Uric Acid | uricase/peroxidase | 135 sec | End Point | 560 nm |
| LDH | pyruvate → lactate | 120 sec | Rate | 340 nm |
| Cholesterol | cholesterol oxidase/ cholesterol esterase/ peroxidase | 135 sec | End Point | 600 nm |
| Triglycerides | lipase/ glycerol kinase/ α-glycerol phosphate dehydrogenase diaphorase | 120 sec | Rate | 500 nm |

*Figure 6.*

TYPICAL STRIP TEST PRECISION

| BUN | | | |
|---|---|---|---|
| # of Runs | 50 | 50 | 50 |
| Mean (mg/dl) | 10.2 | 20.3 | 29.7 |
| Std. Dev. (mg/dl) | 0.60 | 1.15 | 1.32 |
| Coef. of Var. (%) | 5.9 | 5.7 | 4.5 |

| LDH | | | |
|---|---|---|---|
| # of Runs | 20 | 20 | 20 |
| Mean (IU/l) | 173 | 251 | 450 |
| Std. Dev. (IU/l) | 9.5 | 9.9 | 21.0 |
| Coef. of Var. (%) | 5.5 | 4.0 | 4.7 |

| CHOLESTEROL | | | |
|---|---|---|---|
| # of Runs | 26 | 26 | 29 |
| Mean (mg/dl) | 148 | 265 | 382 |
| Std. Dev. (mg/dl) | 8.0 | 14.9 | 16.5 |
| Coef. of Var. (%) | 5.4 | 5.5 | 4.3 |

*Figure 7.*

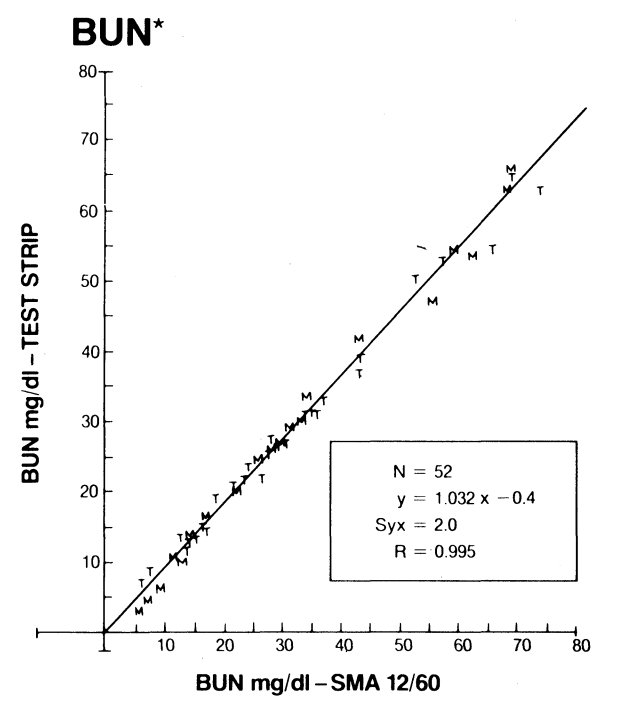

*Differing Symbols denote between day runs

*Figure 8.*

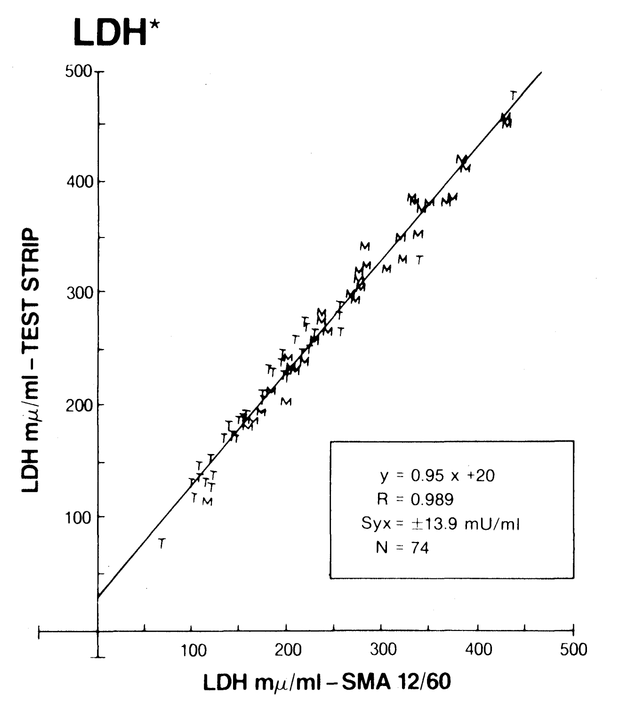

*Differing Symbols denote between day runs

*Figure 9.*

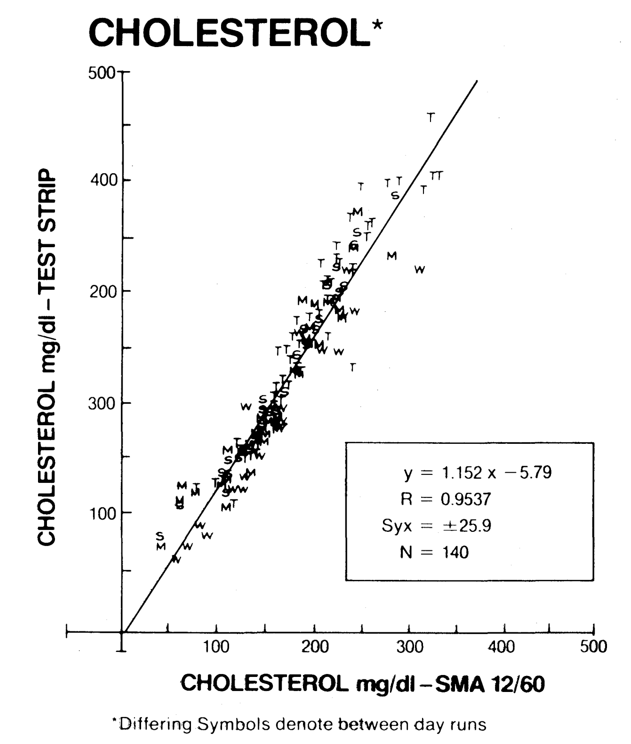

*Figure 10.*

using clinical specimens. Also presented in these figures, are the equations of the regression lines (y=mx +b) as well as the correlation coefficient (R), the number of points in the study (N), and the standard error of the estimate (Syx). The BUN test correlation data which are shown in Figure 8 indicate excellent correlation with the SMA 12/60 with slopes near 1 and a correlation coefficient exceeding 0.99. The LDH data are shown in Figure 9 and similar conclusions can be drawn. Finally, the cholesterol correlation data are shown in Figure 10 and also indicate excellent performance versus the reference method.

Correlations with reference methods are excellent and the C.V.'s obtained by means of these dry strips are comparable to other methodology currently considered acceptable by the medical community. Standard methods have also been compared against one another and the correlations curves were, in general, similar to those achieved with the strip tests. It is concluded, that this technology is highly promising. However, it must be emphasised that it is promising only if the systems are dry and self-contained. Since they offer no significant improvement in analytical capability, the dry systems' major advantages are convenience, simplicity and storageability. However, these are highly significant advantages and the authors are working to achieve a more complete battery of tests. In conclusion, this and related technology represent the imminent future of clinical chemical analyses.

REFERENCES

[1] Ross, J. and Frazer, M.D., (1979) *Am. J. Clin. Path.* 72, 265-273.

### Notes for Contributors

### Presentation of manuscripts

Manuscripts should be typed (double-spaced) on one side of the paper only and with generous margins. The title should be brief and informative avoiding the word "new" and its synonyms. The full list of authors with their affiliations and full address(es) should appear on the title page. On a separate sheet an abstract of no more than 150 words is required. This should succinctly describe the scope of the contribution and highlight significant findings or innovations. It should be written in a style which can easily be translated into French and German.

*The Concise Oxford Dictionary* and Fowler's *Modern English Usage* (both published by Oxford University Press) should be used as the standard for spelling and grammar. Abbreviations should be limited to those generally recognised, or where a frequently occuring term is abbreviated it should, in the first instance, be explained thus "flow injection analysis (FIA) ..." and the abbreviation used thereafter. Abbreviations, for standard measures and units should follow SI recommendations. There are various publications giving guidance on the use of SI units.

References should be indicated in the text by numerals following the author's name, i.e. Skeggs [6]. On a separate sheet of paper, list all references in numerical order thus:

[6] Skeggs, L.T., *American Journal of Clinical Pathology,* 1959, **28**, 311.

Note that journal titles are given in full. Where there is more than one author, the form Foreman et al. should be used in the text but all authors should be named in the list of references. When reference is made to a chapter in a book the reference should take the following form:

[7] Malmstadt, H.V. in "Topics in Automatic Chemistry" Ed. Stockwell P.B. and Foreman J.K. 1978 Horwood, Chichester, pp. 68-70.

Only work which has been published or has been accepted for publication should be cited. Avoid the citation of documents which are subject to restricted circulation, patent literature, unpublished work and personal communications. The latter can be mentioned in the text in parenthesis.

To illustrate a paper line diagrams are preferred to photographs. Photographs should only be used when they significantly add to the discussion. Diagrams, charts and graphs should be carefully drawn in black ink on stout card or heavy quality tracing paper. Most illustrations are reduced for publication; to allow for this originals should be between 16 and 36 cm wide (the depth must not exceed 50 cm). The lettering of diagrams should be sufficiently clear to withstand reduction. Except in the case of proper names, all lettering should be in lower case print. If photographs are used they must be supplied in the form of clear, unmounted, glossy, black and white prints. "Instant" photographs are not normally acceptable. All illustrations must be identified on the reverse showing the figure number and the author's name.

Each illustration should have a fully explanitory caption. Captions should be typed together on a separate sheet of paper; they must *not* be an inseparable part of the illustration.

